# Development and validation of a prognostic nomogram model incorporating routine laboratory biomarkers for preoperative patients with endometrial cancer

**DOI:** 10.1186/s12885-023-11497-8

**Published:** 2023-11-29

**Authors:** Rong Cong, Mingyang Li, Wan Xu, Xiaoxin Ma, Shuhe Wang

**Affiliations:** 1 Department of Obstetrics and Gynecology, the Seventh Medical Center of PLA General Hospital, Beijing, China; 2 Department of Orthopedics, the Fourth Medical Center of PLA General Hospital, Beijing, China; 3 Department of Obstetrics and Gynecology, Shengjing Hospital of China Medical University, Shenyang, China

**Keywords:** Endometrial cancer, Nomogram, External validation, Overall survival, Progression-free survival, Biomarkers

## Abstract

**Background:**

Some biomarkers collected from routine laboratory tests have shown important value in cancer prognosis. The study aimed to evaluate the prognostic significance of routine laboratory biomarkers in patients with endometrial cancer (EC) and to develop credible prognostic nomogram models for clinical application.

**Methods:**

A total of 727 patients were randomly divided into a training set and a validation set. Cox proportional hazards models were used to evaluate each biomarker’s prognostic value, and independent prognostic factors were used to generate overall survival (OS) and progression-free survival (PFS) nomgrams. The efficacy of the nomograms were evaluated by Harrell’s concordance index (C-index), receiver operating characteristic (ROC) curves, decision curve analysis (DCA), calibration curves, X-tile analysis and Kaplan‒Meier curves.

**Results:**

Ten significant biomarkers in multivariate Cox analysis were integrated to develop OS and PFS nomograms. The C-indices of the OS- nomogram in the training and validation sets were 0.885 (95% confidence interval (CI), 0.810–0.960) and 0.850 (95% CI, 0.761–0.939), respectively; those of the PFS- nomogram in the training and validation sets were 0.903 (95% CI, 0.866–0.940) and 0.825 (95% CI, 0.711–0.939), respectively. ROC, DCA and calibration curves showed better clinical application value for the nomograms incorporating routine laboratory biomarkers. X-tile analysis and Kaplan‒Meier curves showed that the nomograms were stable and credible in evaluating patients at different risks.

**Conclusions:**

Nomogram models incorporating routine laboratory biomarkers, including NLR, MLR, fibrinogen, albumin and AB blood type, were demonstrated to be simple, reliable and favourable in predicting the outcomes of patients with EC.

**Supplementary Information:**

The online version contains supplementary material available at 10.1186/s12885-023-11497-8.

## Introduction

Endometrial cancer (EC) is one of the most common gynaecologic malignancies worldwide, with an estimated 66,200 new cases and 13,030 deaths in the United States in 2023 [1]. Despite efforts to combat the disease, the incidence of EC has increased by over 50% in the past 20 years [2]. Approximately 70% of patients with EC are diagnosed at early stages due to the first symptom of uterine bleeding, and their 5-year overall survival (OS) might reach 80% [3]. However, asymptomatic and advanced-stage patients have higher risk. Patients with metastatic disease, pelvic recurrence or extrapelvic recurrence further decrease the 5-year OS rates to 16%, 55% and 17%, respectively [4, 5]. Accurate prediction and reproducible prognostication are therefore crucial for optimal treatment and outcomes.

Traditionally, tumour type, grade and stage are considered the most important prognostic factors in clinical evaluation. In recent years, many clinical tests and pathologic features following ESGO/ESTRO/ESP guidelines have provided clinicians with beneficial prognostic information and treatment recommendations [6]. However, extensive testing increases the economic burden on patients and may not be suitable for long-term surveillance in undeveloped areas. Clinicians often overlook the potential of low-cost and reproducible biomarkers obtained from routine laboratory tests for cancer prognosis. In our previous study, we found that an elevated preoperative blood neutrophil-lymphocyte ratio (NLR), platelet-lymphocyte ratio (PLR), monocyte-lymphocyte ratio (MLR) and fibrinogen levels were significantly associated with poor OS in EC [7, 8]. In subsequent studies, we examined standard tests used in clinical practice, including routine blood analysis, blood lipids, coagulation profiles, liver function indices, renal function indices and ABO blood type, aiming to incorporate these prognostic factors into an accurate and timely prediction model to improve patient outcomes.

Nomograms, which are being increasingly used as prognostic models in oncology [9, 10], can graphically integrate diverse prognostic factors and provide a simplified and personalized scoring system for each patient [11]. In this study, we first developed nomograms using a series of clinicopathological factors to predict risk of PFS and OS in untreated EC patients. Then we performed external validation to verify that the nomogram models integrating routine laboratory biomarkers are simple, reliable and effective in predicting outcomes for EC patients.

## Materials and methods

### Study population

We retrospectively extracted data from Shengjing Hospital of China Medical University databases for EC patients who underwent surgical treatment between December 2016 and December 2018. The inclusion criteria were as follows: (1) patients who underwent gynaecological and imaging examinations and were histologically diagnosed with primary EC; and (2) patients who underwent comprehensive primary surgical treatment, including total hysterectomy with or without bilateral salpingo-oophorectomy and lymphadenectomy. The exclusion criteria were as follows: (1) patients without complete clinicopathological data or lack of continuous follow-up; (2) patients treated with radiotherapy or chemotherapy prior to hysterectomy; (3) patients with other simultaneous malignancies; and (4) patients with coexistent haematologic disorders.

### Data collection

We collected clinical records, including age, Federation International of Gynaecology and Obstetrics (FIGO) stage, tumour grade, histopathological subtype, myometrial invasion depth, lymphovascular space invasion, body mass index and laboratory test results such as neutrophil count, lymphocyte count, monocyte count, platelet count, fibrinogen level, albumin level, triglyceride (TG) level, and high-density lipoprotein cholesterol (HDL-c) level. OS and PFS were selected as the primary and secondary endpoints regarding clinical outcomes. OS was calculated from the date of total hysterectomy to death from any cause. PFS was calculated from the date of total hysterectomy until radiological evidence or biopsies of tumour progression. PFS was censored at the date of death from any cause or the date of the last follow-up visit for progression-free patients.

### Nomogram development

To develop a well-calibrated and effective nomogram, we randomly assigned two-thirds of the patients in the model to a training set and the others to an external validation set using the R function ‘createDataPartition‘ [12, 13]. The last follow-up assessment for the training cohort was performed in May 2020; the last follow-up assessment for the validation cohort was performed in June 2021. For the training set, receiver operating characteristic (ROC) curves were first used to reveal associations between clinicopathological factors and EC prognosis and identify the optimal cut-off of each factor by calculating the maximum area under the curve (AUC). Schoenfeld residuals tests were then used to determine whether the risks associated with EC were constant with survival time. If a P value was lower than 0.05, the variable was considered to be associated with survival time and was included in Cox proportional hazard models. Multicollinearity among clinical variables was evaluated with variance inflation factors (VIFs). A VIF < 5 indicated low collinearity, and the variable could then be included in Cox proportional hazard models. In Cox models, univariable and multivariable analyses were conducted to evaluate each factor’s hazard ratio (HR) and 95% confidence interval (95% CI). Next, possible prognostic factors were used to establish nomograms to predict 3-year and 5-year PFS and OS. Each selected factor was assigned a corresponding score, with the total score applied for individual prediction of PFS and OS for each EC patient. The model efficacy for predicting the prognosis of EC was evaluated with Harrell’s concordance index (C-index) and the AUC of ROC curves analysis. DCA was performed to illustrate whether the nomogram provides valuable and profitable analysis. The discriminative capacity between the outcomes predicted by the nomograms and observed in clinical studies detected with calibration curves. All patients were regrouped into low-, moderate- and high-risk groups according to the total scores of the nomograms. X-Tile 3.6.1 software (Yale School of Medical, USA) was used to calculate the margins of the groups. Kaplan–Meier curves were generated to assess risks for different groups. For external validation, we used the dataset to detect the efficiency of the nomograms and performed additional C-index, ROC and calibration curve analyses for verification.

### Statistical analysis

The ROC curves, multicollinearity analysis, Schoenfeld residuals tests and Cox models were performed using IBM SPSS 19.0 (SPSS, Inc., Chicago, Illinois, USA). R 4.0.2 software (http://www.Rproject.org) was used to develop the nomograms. All statistical tests were two-sided. A P value < 0.05 was considered to denote significant differences.

## Results

### Demographics and clinical characteristics of the patients

**Fig. 1:**
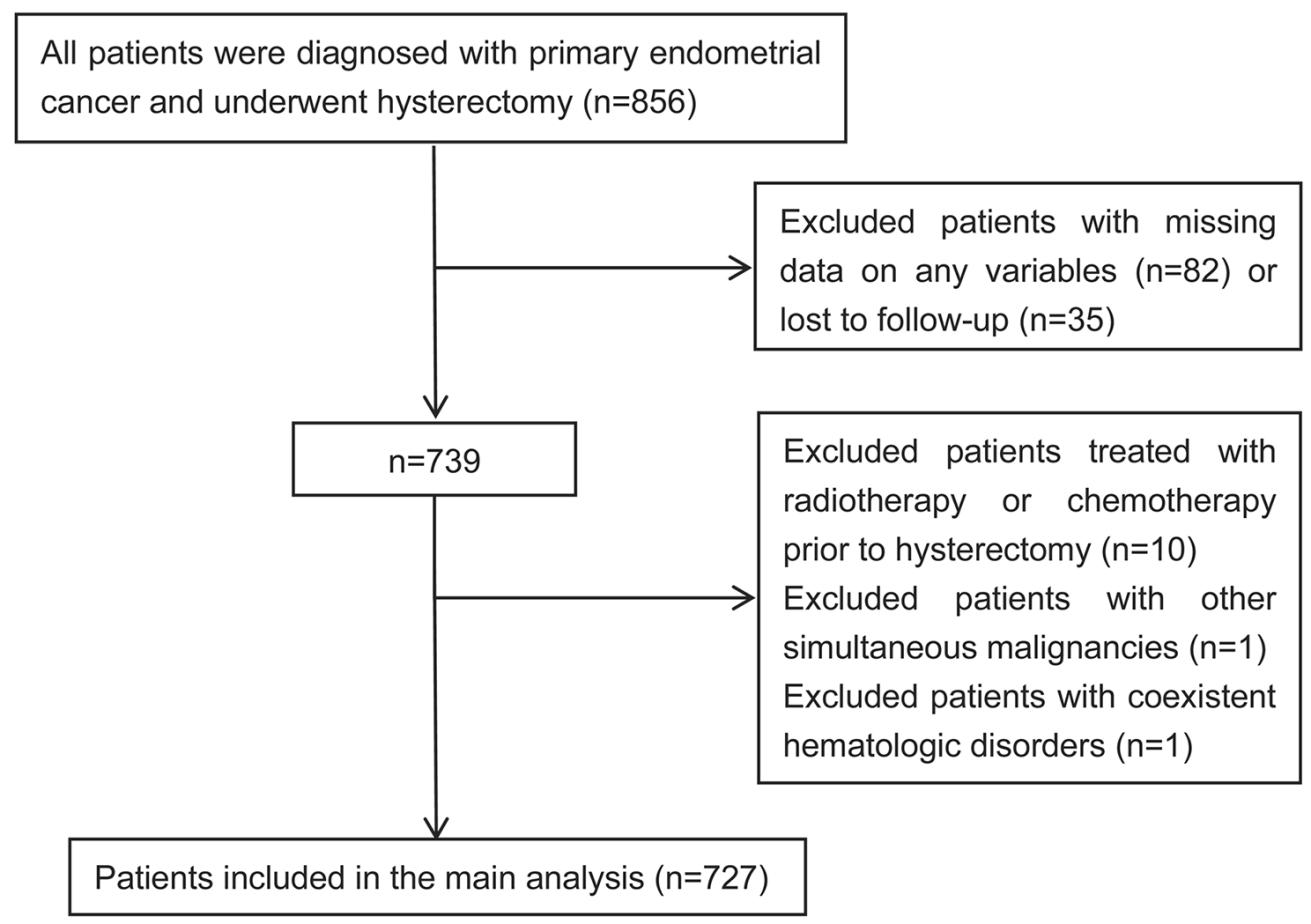
The inclusion and exclusion process

A total of 727 patients with EC who met the inclusion and exclusion criteria were included. The screening process is shown in Fig. 1. The demographics and clinical characteristics of the patients can be found in Table S1. Of the patients, 484 patients (66.6%) were assigned to the training set; 243 patients (33.4%) were assigned to the validation set. There were no significant differences in indicators between the two sets (all P values > 0.05). The median age for both the training and validation sets was 56, with an interquartile range (IQR) of 51–61 for the training set and 49–62 for the validation set. The median follow-up duration was 46 months for the training set (IQR 31–60) and 44 months for the validation set(IQR 30–60). At the last follow-up, 43 (8.9%) patients in the training set and 20 (8.2%) patients in the validation set were found to have confirmed disease progression, including 25 deaths in the former and 15 deaths in the latter.

### Independent prognostic factors in EC

**Fig. 2:**
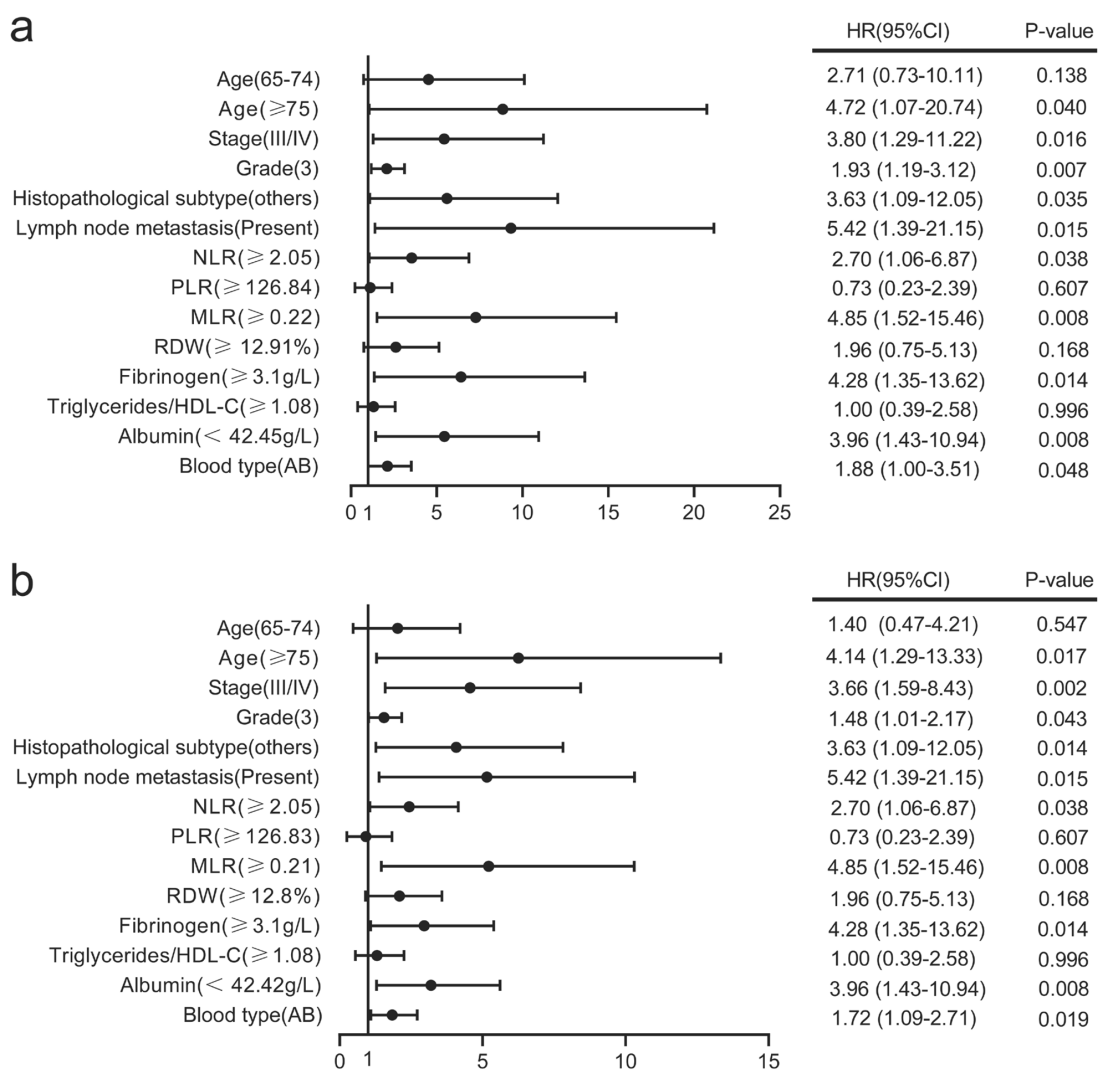
The multivariate Cox analysis of the routine laboratory biomarkers (a) overall survival (b) progression-free survival

Table S2 presents the ROC curve results, showing each indicator’s prediction value, which were used to determine the cut-off for significant indicators. The biomarkers NLR, PLR, MLR, RDW, fibrinogen, TG/HDL-c, and albumin were found to be statistically significant, and the cut-offs for these indicators were 2.05, 126.84, 0.22, 12.91, 3.10, 1.08 and 42.45 for OS, and 2.05, 126.83, 0.21, 12.80, 3.10, 1.08 and 42.42 for PFS, respectively. Patients were then divided into two groups based on these cut-offs. Clinical reference values were used for the other indicators to group the patients in further analyses. Tables S3 and S4 display the results of Schoenfeld residuals and multicollinearity, indicating low collinearity and multicollinearity among the variables. Therefore, all variables were included in the Cox proportional hazard models.According to the results of univariate Cox analyses shown in Tables S5 and S6, age, stage, histopathological subtype, lymph node metastasis, NLR, PLR, MLR, RDW, fibrinogen, TG/HDL-c, albumin and blood type were significantly associated with both OS and PFS. These variables were further used in multivariate Cox analysis (Fig. 2), and age, stage, histopathological subtype, lymph node metastasis, NLR, MLR, fibrinogen, albumin and blood type were significantly associated with both OS and PFS. However, multivariate Cox analysis did not show a significant association between PLR, RDW, and TG/HDL-c.

### Construction and evaluation of the nomograms

**Fig. 3:**
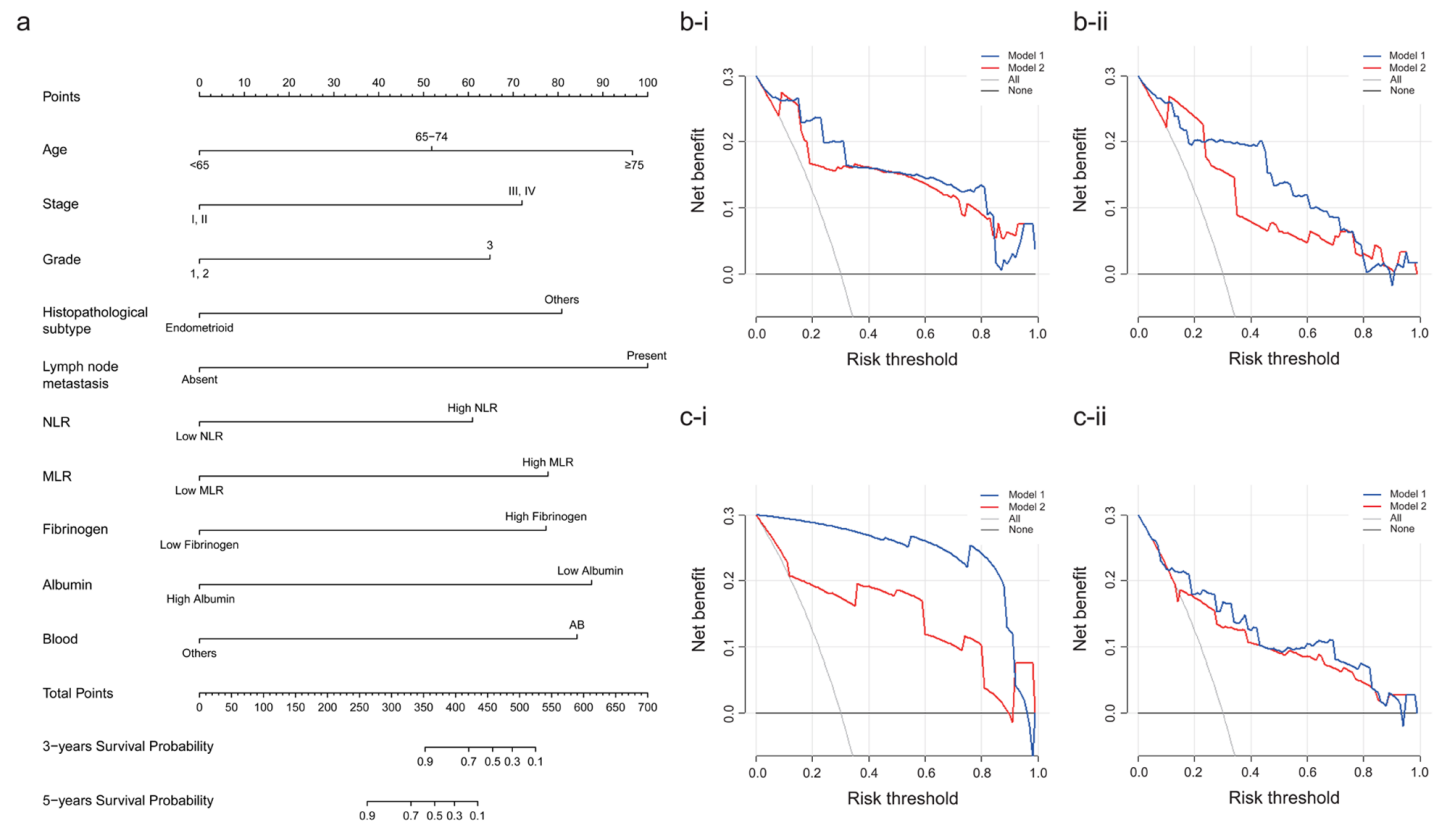
The OS-nomogram of the training set a. The OS-nomogram b-i. 3-year DCA curve of the training set b-ii. 5-year DCA curve of the training set c-i. 3-year DCA curve of the validation set c-ii. 5-year DCA curve of the validation set

**Fig. 4:**
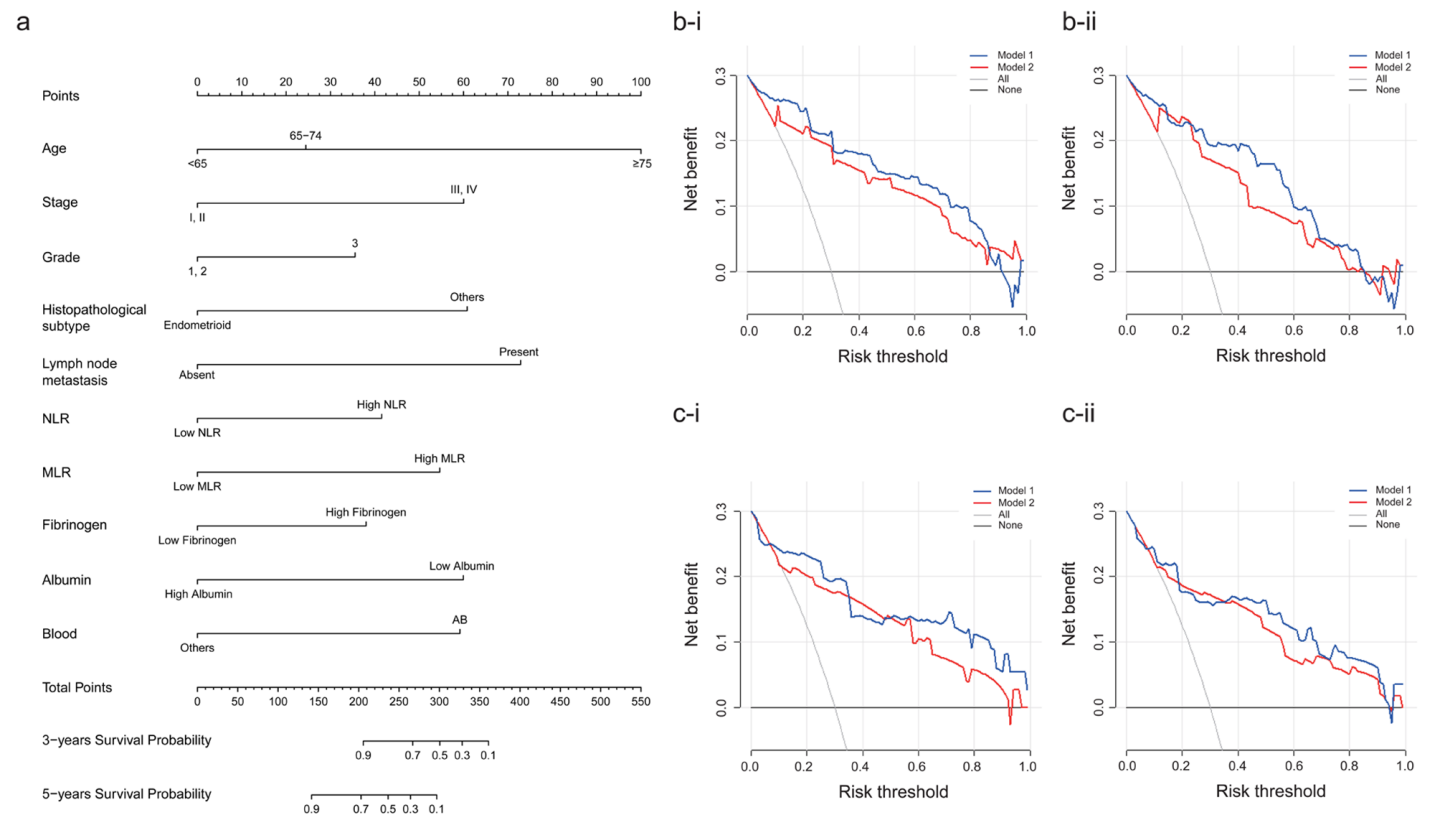
The PFS-nomogram of the training set a. The PFS-nomogram b-i. 3-year DCA curve of the training set b-ii. 5-year DCA curve of the training set c-i. 3-year DCA curve of the validation set c-ii. 5-year DCA curve of the validation set

**Fig. 5:**
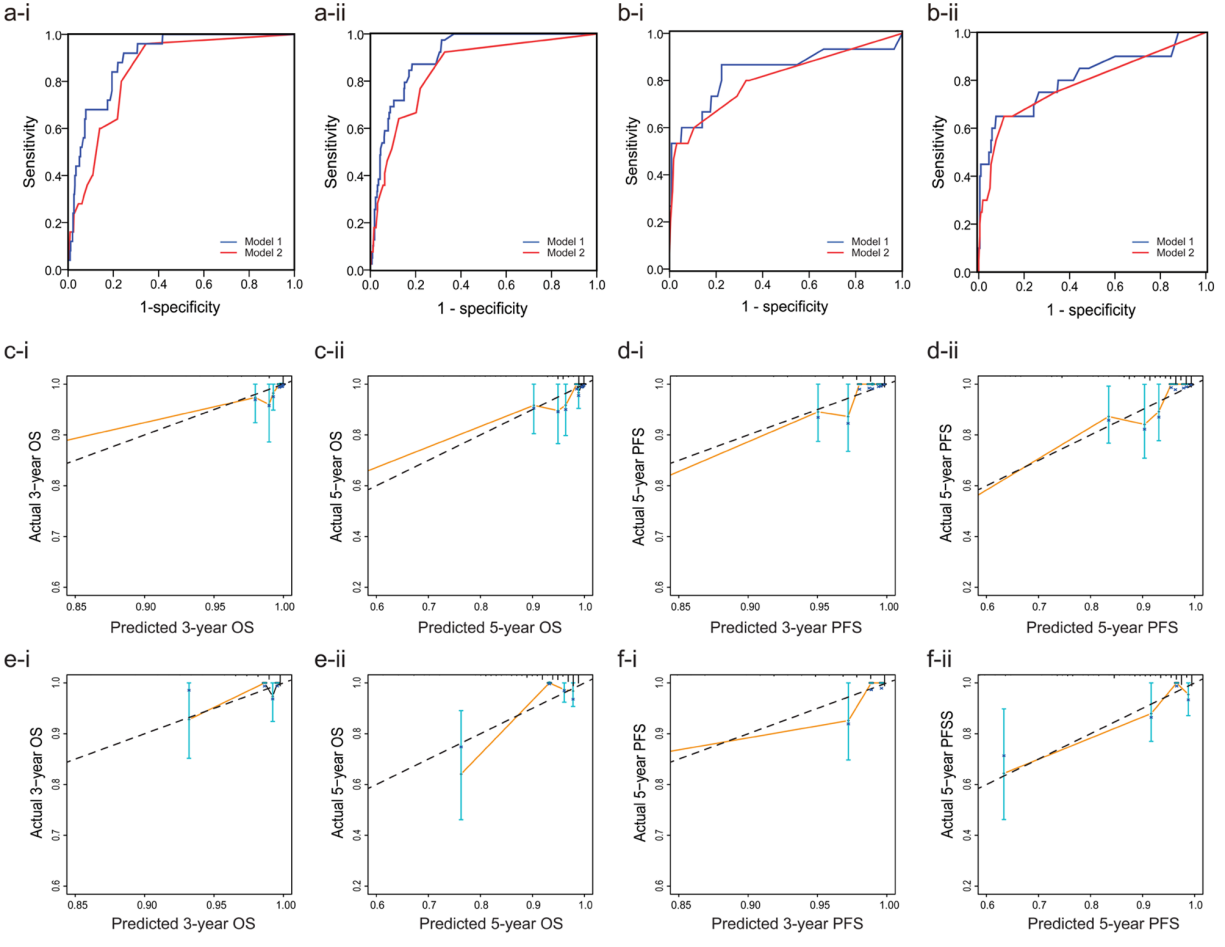
The receiver operating characteristic (ROC) curves and the calibration curves of the nomograms. a-i. The OS-ROC of the training set a-ii. The OS-ROC of the validation set b-i. The PFS-ROC of the training set b-ii. The PFS-ROC of the validation set c-i. 3-year calibration curve of the OS-nomogram of the training set c-ii. 5-year calibration curve of the OS-nomogram of the training set d-i. 3-year calibration curve of the PFS-nomogram of the training set d-ii. 5-year calibration curve of the PFS-nomogram of the training set e-i. 3-year calibration curve of the OS-nomogram of the validation set e-ii. 5-year calibration curve of the OS-nomogram of the validation set f-i. 3-year calibration curve of the PFS-nomogram of the validation set f-ii. 5-year calibration curve of the PFS-nomogram of the validation set

Based on multivariate Cox analysis, ten significant independent prognostic factors were integrated to develop OS and PFS nomogram. As shown in Fig. 3a, lymph node metastasis contributed the most to the OS in EC, followed by ages; as indicated in Fig. 4a, age had the most significant influence on PFS, followed by lymph node metastasis. The C-indices of the OS- nomogram in the training and validation sets were 0.885 (95% CI, 0.810–0.960) and 0.850 (95% CI, 0.761–0.939), respectively, and those of the PFS- nomogram in the training and validation sets were 0.903 (95% CI, 0.866–0.940) and 0.825 (95% CI, 0.711–0.939), respectively. We then reexamined the ROC curves to compare the efficacy of the nomograms with and without the indicators NLR, MLR, fibrinogen, albumin, and blood type(Fig. 5a-b). The AUC of the OS- nomogram in Model 1(including the indicators) was 0.898 (95% CI, 0.852–0.943), and that of the PFS- nomogram was 0.907 (95% CI, 0.872–0.943). In contrast, in Model 2 (excluding the indicators), the values declined to 0.845 (95% CI, 0.782–0.907) and 0.848 (95% CI, 0.787–0.908), respectively. In the validation set, the AUC of the OS-nomogram in Model 1 was 0.838 (95% CI, 0.700–0.976), and that in Model 2 was 0.808 (95% CI, 0.669–0.946).The AUC of the PFS-nomogram in Model 1 was 0.809 (95% CI, 0.687–0.931), and that in Model 2 was 0.784 (95% CI, 0.656–0.912).

The 3-year and 5-year DCAs of the OS-nomograms in the training set are shown in Fig. 3b. Model 1 generally had better net benefits than Model 2 in both analyses. Similar findings were observed for the validation set (Fig. 3c). The PFS nomograms also showed similar trends in DCA analyses (Fig. 4b-c). The calibration curves for risk of progression or death within 3 and 5 years in the training and validation sets are shown in Fig. 5c-f. All curves demonstrated optimal agreement between the predicted and observed lines.

**Fig. 6:**
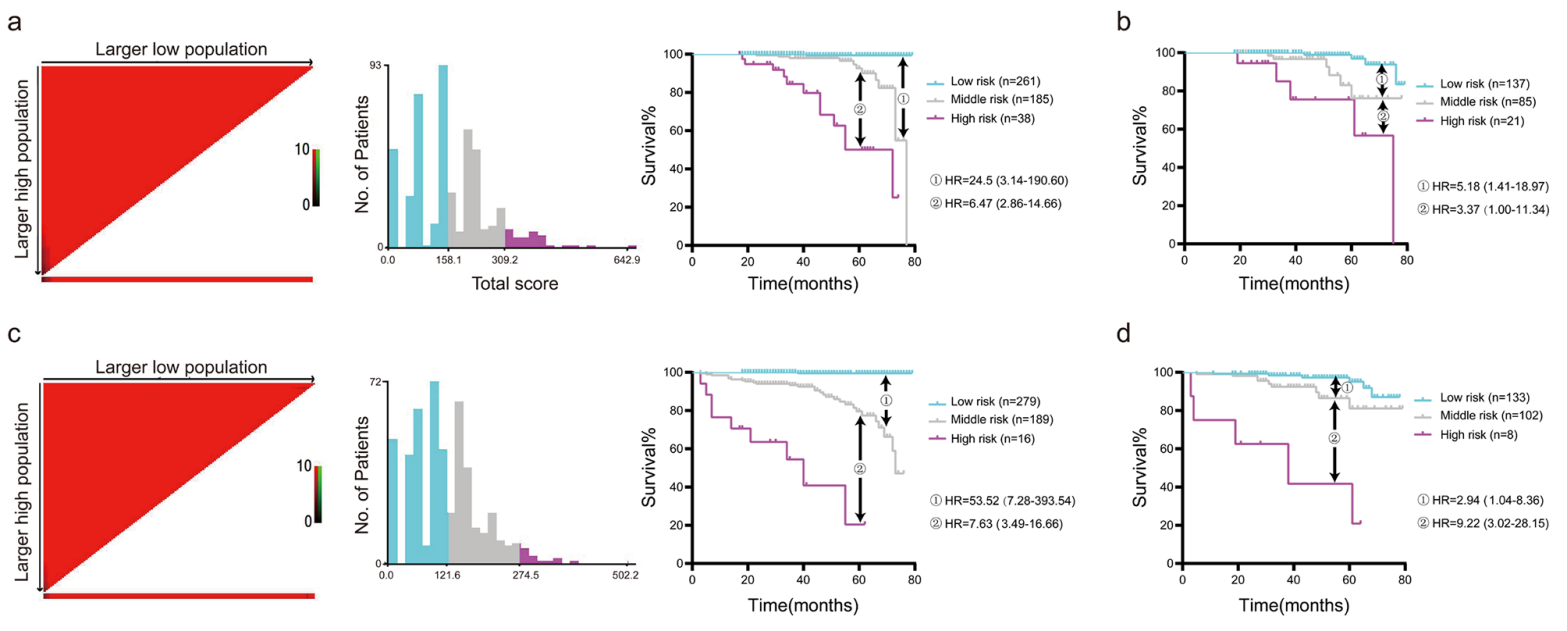
X-tile analysis and Kaplan-Meier curves of the nomograms a X-tile analysis of the total risk score and Kaplan-Meier curves about the overall survival in the training set b Kaplan-Meier curves about the overall survival in the validation set c X-tile analysis of the total risk score and Kaplan-Meier curves about the progression-free survival in the training set d Kaplan-Meier curves about the progression-free survival in the validation set

The distributions of total risk scores of the nomograms and the cut-offs detected by X-tile analysis are displayed in Fig. 6a,c. For the OS- nomogram, risks were divided into low risk (total score < 158.1), moderate risk (158.1 < total score < 309.2) and high risk (total score > 309.2). For the PFS- nomogram, risks were divided into low risk (total score < 121.6), moderate risk (121.6 < total score < 274.5) and high risk (total score > 274.5). Patients with lower total scores generally had lower risk and better OS and PFS than those with higher scores. The Kaplan‒Meier curves showed that for OS, the hazard ratios (HRs) for the moderate and high-risk categories were 24.5 (95% CI, 3.14–190.60) and 6.47 (95% CI, 2.86–14.66), respectively, compared to the low or moderate-risk category. For PFS, HRs were 53.62 (95% CI, 7.28–393.54) and 7.63 (95% CI, 3.49–16.66), respectively. Similar trends were observed for the validation set (Fig. 6b, d), in which patients with lower total scores generally had lower risk and better OS and PFS.

## Discussion

Preoperative risk stratification is one of the greatest challenges in EC treatment [14]. Conventional FIGO staging systems have limitations for predicting the prognosis of EC after surgery [15]. Routine laboratory tests are simple and convenient for doctors to evaluate prognosis and are minimally invasive and cost-effective for patients [16]. Therefore, establishing an objective predictive model incorporating laboratory indicators would be meaningful for evaluating the prognosis of EC. Nomogram model have reasonable and personalized prognostic value in facilitating management-related decisions [17], integrating multiple clinical and biological risk factors into the evaluation and providing a visual, objective, and individualized scoring system for each patient [11]. To verify our model’s predictive power, we established an independent validation set of patients. This method ensures the model’s generalizability for wide use in different patient populations [18].

The nomogram developed in this study demonstrated excellent performance in predicting PFS and OS for patients with EC who had undergone comprehensive primary surgical treatment with total hysterectomy. Indeed, calibration curves displayed good discriminatory power in predicting 3-year and 5-year PFS and OS. The C-index and AUC in the training and validation sets indicated that the nomogram incorporating NLR, MLR, fibrinogen, albumin, and ABO blood type into the scoring system was reliable at predicting EC prognosis. The DCA for Model 1 also showed incredible net benefits, indicating that the nomogram involving the above indicators has good predictive power and clinical utility. Additionally, all calibration curves showed little deviation from the reference line, indicating high credibility. Thus, routine laboratory indicators are necessary for evaluating the outcomes of patients with EC. Furthermore, we categorized patients into three risk groups based on the prognostic nomogram scores using X-tile analysis. The findings revealed the risks associated with different scores and can help clinicians to intuitively predict the probability of survival.

This study examined a series of indicators collected from standard blood tests. Eventually, we found that high NLR, high MLR, high RDW, high fibrinogen, low albumin, and type AB blood were significantly associated with poor OS and PFS in EC. Our previous study described that tumour progression and systemic inflammatory responses disrupt the balance among routine blood constituents [7]. Tumour cells induce an increase in neutrophils and monocytes, and in turn, neutrophils and monocytes inhibit the antitumour immune response [19]. In contrast, tumour-infiltrating lymphocytes exhibit potent antitumour functions [20]. Thus, increased NLR and MLR reflect the host’s immune status and might be associated with tumour progression and poor outcomes, as supported by similar findings in other malignancies [21]. Fibrinogen and albumin are two acute-phase proteins induced in response to systemic inflammation but show opposite abundance trends under cancer inflammatory stimulation [22]. Tumour cells directly synthesize fibrinogen and produce interleukin-6 to stimulate fibrinogen secretion, leading to tumour progression and metastasis [23]. Albumin is an essential protein for nutrition transport and body metabolism, and hypoalbuminaemia is associated with poor outcomes in various tumours [16]. Our previous study revealed that high fibrinogen and low albumin are significant prognostic factors for EC patients [8]. The ABO blood group is also associated with EC prognosis. One hypothesis suggests that under normal conditions, von Willebrand factor (vWF) stabilizes factor VIII (FVIII) and transports it to injury sites, interacting with platelets and promoting the clotting process. The function of vWF is partly regulated by metalloprotease, which clears vWF from the plasma. The A and B antigens interfere with cleavage sites, reducing clearance of vWF. People with the AB blood group have the highest levels of vWF and FVIII in their plasma, which puts them at the highest risk of venous and arterial thromboembolism [24]. Additionally, existing evidence suggests that cancers are often associated with a hypercoagulability state [25]. These findings are consistent with many published studies showing a close association between the prognosis of various cancers and patient nutrition and immune status [26]. The underlying mechanism may be that chronic systemic inflammation in patients depletes available nutrition and energy, leading to hypoalbuminaemia or even cachexia, resulting in poor outcomes [27].

In some cases, PLR, RDW, and TG/HDL-c have been recommended as prognostic markers for EC [28–30]. However, after considering dozens of routine laboratory biomarkers, they were found to be significantly associated with OS and PFS in univariate analysis but not in multivariate analysis. The prognostic values of those markers may not be sufficient when considered together, which is why they were not significant in multivariate analysis. Nevertheless, we cannot deny their association with EC prognosis, and it is essential to be cautious if their values are abnormal. Overall, the significant biomarkers identified in multivariate analysis deserve more attention to establish a precise and reliable prognostic evaluation system.

To the best of our knowledge, this nomogram model integrates the most comprehensive laboratory biomarkers in China. The findings are in line with existing studies and the model performs good robustness via external validation. However, there are still some weakness. First, as this study focuses on routine laboratory biomarkers, some prognostic markers were not included, such as tumour sizes and blood group antigens [31, 32]. In addition, histopathological evaluation has a strong association with EC prognosis and is generally taken as a cornerstone for EC classification. The ESTRO/ESGO/ESP proposes four molecular TCGA molecular groups, namely, POLE ultramutated (POLEmut), mismatch repair-deficient (MMRd), p53 mutant (p53abn), and others referred to as NSMP (non‐specific molecular profile), to assess the prognosis of EC [6, 33]. In some cases, molecular subgroups have been integrated into prognostic models to evaluate the association of other clinicopathologic factors with EC [34]. It’s a pity that molecular marker detection was not extensively used in our institution during data collection. The nomogram is an inclusive model, and we will integrate more clinicopathologic factors to make the model more precise. Secondly, although we made external validation to mimic new patient cohorts, it is still a single-institution study. We will unite more institutions to improve the application universality and prediction accuracy.

## Conclusions

In conclusion, we developed and validated an OS-nomogram and a PFS-nomogram for EC patients by incorporating routine laboratory markers, NLR, MLR, RDW, fibrinogen, albumin and AB blood type. The models generated were demonstrated to be simple, reliable and favourable for predicting the outcomes of patients with EC.

### Electronic supplementary material

Below is the link to the electronic supplementary material.

Supplementary Material 1

Supplementary Material 2

Supplementary Material 3

Supplementary Material 4

Supplementary Material 5

Supplementary Material 6

## Data Availability

The data supporting this study’s findings are available from the corresponding author upon reasonable request.

## Abbreviations

EC: endometrial cancer
OS: overall survival
PFS: progression-free survival
C-index: Harrell’s concordance index
ROC: receiver operating characteristic curves
DCA: decision curve analysis
CI: confidence interval
CA125: cancer antigen 125
HE4: human epididymis protein 4
HE4: neutrophil-lymphocyte ratio (x)
PLR: platelet-lymphocyte ratio
MLR: monocyte-lymphocyte ratio
TG: triglyceride
HDL-c: high-density lipoprotein cholesterol
AUC: area under the curve
VIF: variance inflation factors
HR: hazard ratio
RDW: red cell distribution width
IQR: interquartile range
vWF: Von Willebrand factor
FVIII: factor VIII
