# Supplementary material for: Development and validation of a prognostic nomogram model incorporating routine laboratory biomarkers for preoperative patients with endometrial cancer
Source: BMC Cancer. 2023 Nov 29;23:1167. doi: 10.1186/s12885-023-11497-8 (PMC10688010; doi:10.1186/s12885-023-11497-8)
Supplement: Supplementary file 1 — Supplementary Material 1 [file 12885_2023_11497_MOESM1_ESM.docx]

| Clinicopathologic Characteristics | Total (n= 727) | Primary cohort (n= 484) | Validation cohort (n= 243) | P |
| --- | --- | --- | --- | --- |
|  | No (%) | No (%) | No (%) |  |
| Age, y |  |  |  |  |
| ＜65 | 630 (86.7) | 423 (87.4) | 207 (85.2) | 0.699 |
| 65-74 | 80 (11.0) | 50 (10.3) | 30 (12.3) |  |
| ≥75 | 17 (2.3) | 11 (2.3) | 6(2.5) |  |
| Stage |  |  |  |  |
| I, II | 659 (90.6) | 442 (91.3) | 217(89.3) | 0.377 |
| III, IV | 68 (9.4) | 42 (8.7) | 26 (10.7) |  |
| Grade |  |  |  |  |
| 1, 2 | 580 (79.8) | 384 (79.3) | 196 (80.7) | 0.676 |
| 3 | 147 (20.2) | 100 (20.7) | 47 (19.3) |  |
| Histopathological subtype |  |  |  |  |
| Endometrioid | 677 (93.1) | 451 (93.2) | 226 (93.0) | 0.578 |
| Others | 50 (6.9) | 33 (6.8) | 17 (7.0) |  |
| Lymph node metastasis |  |  |  |  |
| Absent | 696 (95.7) | 465 (96.1) | 231 (95.1) | 0.524 |
| Present | 31 (4.3) | 19 (3.9) | 12 (4.9) |  |
| NLR |  |  |  |  |
| ＜2.05 | 393 (54.1) | 265 (54.8) | 128 (52.7) | 0.596 |
| ≥2.05 | 334 (45.9) | 219 (45.2) | 115 (47.3) |  |
| MLR |  |  |  |  |
| Low group (＜0.22 for OS) | 465 (64.0) | 306 (63.2) | 159 (65.4) | 0.558 |
| High group (≥0.22 for OS) | 262 (36.0) | 178 (36.8) | 84 (34.6) |  |
| MLR |  |  |  |  |
| Low group (＜0.21 for PFS) | 309 (42.5) | 305 (63.1) | 159 (65.4) | 0.523 |
| High group (≥0.22 for PFS) | 418 (57.5) | 179 (36.9) | 84 (34.6) |  |
| Fibrinogen, g/L |  |  |  |  |
| ＜3.1 | 435 (59.8) | 284 (58.7) | 151 (62.1) | 0.369 |
| ≥3.1 | 292 (40.2) | 200 (41.3) | 92 (37.9) |  |
| Albumin, g/L |  |  |  |  |
| High group (≥42.45 for OS) | 464 (63.8) | 314 (64.9) | 150 (61.7) | 0.405 |
| Low group (＜42.45 for OS) | 263 (36.2) | 170 (35.1) | 93 (38.3) |  |
| Albumin, g/L |  |  |  |  |
| High group (≥42.42 for PFS) | 463 (63.7) | 313 (64.7) | 150 (61.7) | 0.437 |
| Low group (＜42.42 for PFS) | 264 (36.3) | 171 (35.3) | 93 (38.3) |  |
| Blood type |  |  |  |  |
| Others | 702 (96.6) | 468 (96.7) | 234 (96.3) | 0.781 |
| AB | 25 (3.4) | 16 (3.3) | 9 (3.7) |  |

**Table S1** Patient demographics and clinical characteristics
