# Supplementary material for: Development and validation of a prognostic nomogram model incorporating routine laboratory biomarkers for preoperative patients with endometrial cancer
Source: BMC Cancer. 2023 Nov 29;23:1167. doi: 10.1186/s12885-023-11497-8 (PMC10688010; doi:10.1186/s12885-023-11497-8)
Supplement: Supplementary file 2 — Supplementary Material 2 [file 12885_2023_11497_MOESM2_ESM.docx]

**Table S2** The AUC of the ROC curves

| Characteristics | OS | | | | | PFS | | | |  |
| --- | --- | --- | --- | --- | --- | --- | --- | --- | --- | --- |
|  | AUC | 95%CI | | P | AUC | | 95%CI | | P | |
| NLR | 0.681 | 0.603 | 0.759 | 0.002 | 0.635 | | 0.544 | 0.725 | 0.005 | |
| PLR | 0.683 | 0.573 | 0.793 | 0.002 | 0.624 | | 0.529 | 0.719 | 0.010 | |
| MLR | 0.790 | 0.692 | 0.888 | ＜0.001 | 0.680 | | 0.582 | 0.778 | ＜0.001 | |
| Peripheral blood eosinophils percentage | 0.453 | 0.360 | 0.545 | 0.425 | 0.480 | | 0.394 | 0.566 | 0.679 | |
| Hemoglobin | 0.493 | 0.387 | 0.599 | 0.908 | 0.457 | | 0.365 | 0.548 | 0.369 | |
| RDW | 0.687 | 0.589 | 0.786 | 0.002 | 0.617 | | 0.524 | 0.710 | 0.015 | |
| Platelet count | 0.517 | 0.400 | 0.634 | 0.772 | 0.571 | | 0.479 | 0.663 | 0.143 | |
| PDW | 0.612 | 0.528 | 0.696 | 0.059 | 0.548 | | 0.465 | 0.632 | 0.318 | |
| PT | 0.502 | 0.390 | 0.614 | 0.974 | 0.539 | | 0.449 | 0.629 | 0.420 | |
| APTT | 0.501 | 0.376 | 0.625 | 0.991 | 0.564 | | 0.468 | 0.660 | 0.185 | |
| PTA | 0.489 | 0.385 | 0.593 | 0.853 | 0.466 | | 0.380 | 0.551 | 0.475 | |
| D-dimer | 0.617 | 0.501 | 0.733 | 0.048 | 0.582 | | 0.481 | 0.683 | 0.089 | |
| Fibrinogen | 0.756 | 0.668 | 0.844 | ＜0.001 | 0.678 | | 0.587 | 0.770 | ＜0.001 | |
| Total cholesterol | 0.560 | 0.447 | 0.673 | 0.314 | 0.570 | | 0.482 | 0.658 | 0.145 | |
| Triglycerides/HDL-C | 0.736 | 0.645 | 0.826 | ＜0.001 | 0.668 | | 0.591 | 0.744 | 0.001 | |
| Albumin | 0.782 | 0.690 | 0.874 | ＜0.001 | 0.671 | | 0.582 | 0.760 | ＜0.001 | |
| AST | 0.476 | 0.365 | 0.587 | 0.688 | 0.472 | | 0.378 | 0.566 | 0.567 | |
| ALT | 0.555 | 0.449 | 0.661 | 0.356 | 0.477 | | 0.387 | 0.566 | 0.628 | |
| ALP | 0.528 | 0.400 | 0.655 | 0.642 | 0.535 | | 0.438 | 0.633 | 0.462 | |
| Creatinine | 0.509 | 0.388 | 0.631 | 0.878 | 0.545 | | 0.449 | 0.642 | 0.348 | |
| Uric acid | 0.544 | 0.421 | 0.667 | 0.458 | 0.534 | | 0.439 | 0.629 | 0.482 | |
