# Supplementary material for: Development and validation of a prognostic nomogram model incorporating routine laboratory biomarkers for preoperative patients with endometrial cancer
Source: BMC Cancer. 2023 Nov 29;23:1167. doi: 10.1186/s12885-023-11497-8 (PMC10688010; doi:10.1186/s12885-023-11497-8)
Supplement: Supplementary file 3 — Supplementary Material 3 [file 12885_2023_11497_MOESM3_ESM.docx]

| Characteristics | OS | | PFS | |
| --- | --- | --- | --- | --- |
|  | pearson correlation | P | pearson correlation | P |
| Age | -0.150 | 0.466 | 0.026 | 0.874 |
| Stage | -0.169 | 0.421 | -0.207 | 0.205 |
| Grade | -0.011 | 0.958 | 0.305 | 0.059 |
| Histopathological subtype | 0.179 | 0.392 | 0.206 | 0.208 |
| Lymph node metastasis | 0.308 | 0.134 | 0.096 | 0.563 |
| NLR | -0.001 | 0.997 | -0.015 | 0.926 |
| PLR | 0.193 | 0.354 | 0.304 | 0.060 |
| MLR | 0.118 | 0.573 | 0.085 | 0.609 |
| Fibrinogen | 0.281 | 0.174 | 0.167 | 0.310 |
| Albumin | 0.048 | 0.819 | 0.015 | 0.929 |
| Triglycerides/HDL-C | -0.040 | 0.849 | 0.016 | 0.921 |
| RDW | 0.125 | 0.552 | 0.073 | 0.659 |
| Blood type | 0.320 | 0.119 | 0.368 | 0.053 |

**Table S3** The results of Schoenfeld residuals
