# Supplementary material for: Development and validation of a prognostic nomogram model incorporating routine laboratory biomarkers for preoperative patients with endometrial cancer
Source: BMC Cancer. 2023 Nov 29;23:1167. doi: 10.1186/s12885-023-11497-8 (PMC10688010; doi:10.1186/s12885-023-11497-8)
Supplement: Supplementary file 4 — Supplementary Material 4 [file 12885_2023_11497_MOESM4_ESM.docx]

**Table S4** The results of multicollinearity

| Characteristics | OS | | PFS | |
| --- | --- | --- | --- | --- |
|  | Tolerance | VIF | Tolerance | VIF |
| Age | 0.919 | 1.088 | 0.929 | 1.076 |
| Stage | 0.842 | 1.187 | 0.835 | 1.197 |
| Grade | 0.903 | 1.108 | 0.896 | 1.116 |
| Histopathological subtype | 0.927 | 1.078 | 0.930 | 1.075 |
| Lymph node metastasis | 0.819 | 1.220 | 0.812 | 1.231 |
| NLR | 0.897 | 1.114 | 0.896 | 1.116 |
| PLR | 0.294 | 3.400 | 0.318 | 3.148 |
| MLR | 0.297 | 3.371 | 0.316 | 3.169 |
| Fibrinogen | 0.893 | 1.120 | 0.900 | 1.112 |
| Albumin | 0.933 | 1.072 | 0.935 | 1.070 |
| Triglycerides/HDL-C | 0.953 | 1.049 | 0.945 | 1.059 |
| RDW | 0.893 | 1.019 | 0.974 | 1.026 |
| Blood type | 0.979 | 1.021 | 0.988 | 1.012 |
