# Supplementary material for: Development and validation of a prognostic nomogram model incorporating routine laboratory biomarkers for preoperative patients with endometrial cancer
Source: BMC Cancer. 2023 Nov 29;23:1167. doi: 10.1186/s12885-023-11497-8 (PMC10688010; doi:10.1186/s12885-023-11497-8)
Supplement: Supplementary file 5 — Supplementary Material 5 [file 12885_2023_11497_MOESM5_ESM.docx]

| Characteristics | Univariate Analysis,  HR (95% CI) | P | Characteristics | Univariate Analysis,  HR (95% CI) | P |  |  |  |  |
| --- | --- | --- | --- | --- | --- | --- | --- | --- | --- |
| Age, y | | | PT, s | | |  |  |  |  |
| ＜65 | 1.00 |  | ＜10.5 | 1.00 |  |  |  |  |  |
| 65-74 | 3.05 (1.01-9.21) | 0.048 | 10.5-13.5 | 0.81 (0.24-2.74) | 0.741 |  |  |  |  |
| ≥75 | 17.75 (5.71-55.21) | ＜0.001 | ＞13.5 | 3.22 (0.33-31.67) | 0.316 |  |  |  |  |
| Stage | | | APTT, s | | |  |  |  |  |
| I, II | 1.00 |  | ≤37 | 1.00 |  |  |  |  |  |
| III, IV | 4.35 (1.86-10.15) | 0.001 | ＞37 | 1.18 (0.28-5.05) | 0.820 |  |  |  |  |
| Grade | | | PTA, % | | |  |  |  |  |
| 1, 2 | 1.00 |  | ＜80 | 1.00 |  |  |  |  |  |
| 3 | 4.38 (2.00-9.61) | ＜0.001 | 80-160 | 43599.95 (0.00-1.04E223) | 0.967 |  |  |  |  |
| Histopathological subtype | | | ＞160 | 356750.04 (0.00-8.61E223) | 0.960 |  |  |  |  |
| Endometrioid | 1.00 |  | D-dimer, ug/L | | |  |  |  |  |
| Others | 4.04 (1.50-10.91) | 0.006 | ≤252 | 1.00 |  |  |  |  |  |
| Lymph node metastasis | | | ＞252 | 2.15 (0.85-5.42) | 0.105 |  |  |  |  |
| Absent | 1.00 |  | Fibrinogen, g/L | | |  |  |  |  |
| Present | 8.61 (3.20-23.18) | ＜0.001 | ＜3.1 | 1.00 |  |  |  |  |  |
| NLR | | | ≥3.1 | 4.87 (2.02-11.78) | ＜0.001 |  |  |  |  |
| ＜2.05 | 1.00 |  | Total cholesterol, mmol/L | | |  |  |  |  |
| ≥2.05 | 2.31 (1.03-5.14) | 0.041 | ＜3.36 | 1.00 |  |  |  |  |  |
| PLR | | | 3.36-5.69 | 23995.97 (0.00-5.50E107) | 0.934 |  |  |  |  |
| ＜126.84 | 1.00 |  | ＞5.69 | 27468.86 (0.00-6.30E107) | 0.933 |  |  |  |  |
| ≥126.84 | 2.32 (1.01-5.33) | 0.047 | Triglycerides/HDL-C | | |  |  |  |  |
| MLR | | | ＜1.08 | 1.00 |  |  |  |  |  |
| ＜0.22 | 1.00 |  | ≥1.08 | 2.30 (1.04-5.10) | 0.040 |  |  |  |  |
| ≥0.22 | 2.52 (1.14-5.57) | 0.023 | Albumin, g/L | | |  |  |  |  |
| Peripheral blood eosinophils percentage , % | | | ≥42.45 | 1.00 |  |  |  |  |  |
| ＜0.7 | 1.00 |  | ＜42.45 | 2.38 (1.08-5.25) | 0.032 |  |  |  |  |
| ≥0.7 | 3.44 (0.46-25.47) | 0.227 | AST, U/L | | |  |  |  |  |
| Hemoglobin, g/L | | | ≤34 | 1.00 |  |  |  |  |  |
| ＜110 | 1.00 |  | ＞34 | 0.56 (0.07-4.13) | 0.566 |  |  |  |  |
| ≥110 | 3.03 (0.40-22.68) | 0.280 | ALT, U/L | | |  |  |  |  |
| RDW, % | | | ≤40 | 1.00 |  |  |  |  |  |
| ＜12.91 | 1.00 |  | ＞40 | 0.95 (0.22-4.05) | 0.946 |  |  |  |  |
| ≥12.91 | 2.32 (1.02-5.27) | 0.044 | ALP, U/L | | |  |  |  |  |
| Platelet count, 10^9/L | | | ≤150 | 1.00 |  |  |  |  |  |
| ＜135 | 1.00 |  | ＞150 | 0.05 (0.00-24697998.16) | 0.768 |  |  |  |  |
| 135-350 | 62039.87 (0.00-7.98E274) | 0.972 | Creatinine, umol/L | | |  |  |  |  |
| ＞350 | 83691.98 (0.00-1.08E275) | 0.972 | ≤84 | 1.00 |  |  |  |  |  |
| PDW, % | | | ＞84 | 2.26 (0.30-16.86) | 0.425 |  |  |  |  |
| ＜11.5 | 1.00 |  | Uric acid, umol/L | | |  |  |  |  |
| 11.5-16.5 | 7119.71 (0.00-9.19E85) | 0.927 | ≤420 | 1.00 |  |  |  |  |  |
| ＞16.5 | 12356.11 (0.00-1.60E86) | 0.922 | ＞420 | 2.27 (0.67-7.67) | 0.186 |  |  |  |  |
| Blood type | | |  |  |  |  |  |  |  |
| Others | 1.00 |  |  |  |  |  |  |  |  |
| AB | 3.20 (1.17-8.76) | 0.023 |  |  |  |  |  |  |  |

**Table S5** Overall survival of the routine laboratory biomarkers with other clinicopathological variables
