# Supplementary material for: Development and validation of a prognostic nomogram model incorporating routine laboratory biomarkers for preoperative patients with endometrial cancer
Source: BMC Cancer. 2023 Nov 29;23:1167. doi: 10.1186/s12885-023-11497-8 (PMC10688010; doi:10.1186/s12885-023-11497-8)
Supplement: Supplementary file 6 — Supplementary Material 6 [file 12885_2023_11497_MOESM6_ESM.docx]

| Characteristics | Univariate Analysis,  HR (95% CI) | P | Characteristics | Univariate Analysis,  HR (95% CI) | P |  |  |  |  |
| --- | --- | --- | --- | --- | --- | --- | --- | --- | --- |
| Age, y | | | PT, s | | |  |  |  |  |
| ＜65 | 1.00 |  | ＜10.5 | 1.00 |  |  |  |  |  |
| 65-74 | 1.86 (0.72-4.84) | 0.203 | 10.5-13.5 | 1.05 (0.37-2.97) | 0.924 |  |  |  |  |
| ≥75 | 12.69 (5.17-31.15) | ＜0.001 | ＞13.5 | 1.95 (0.22-17.58) | 0.550 |  |  |  |  |
| Stage | | | APTT, s | | |  |  |  |  |
| I, II | 1.00 |  | ≤37 | 1.00 |  |  |  |  |  |
| III, IV | 5.25 (2.69-10.26) | ＜0.001 | ＞37 | 1.68 (0.52-5.45) | 0.390 |  |  |  |  |
| Grade | | | PTA, % | | |  |  |  |  |
| 1, 2 | 1.00 |  | ＜80 | 1.00 |  |  |  |  |  |
| 3 | 4.33 (2.31-8.12) | ＜0.001 | 80-160 | 103656.78 (0.00-7.07E248) | 0.968 |  |  |  |  |
| Histopathological subtype | | | ＞160 | 387451.63 (0.00-2.65E249) | 0.964 |  |  |  |  |
| Endometrioid | 1.00 |  | D-dimer, ug/L | | |  |  |  |  |
| Others | 3.54 (1.56-8.03) | ＜0.001 | ≤252 | 1.00 |  |  |  |  |  |
| Lymph node metastasis | | | ＞252 | 1.69 (0.78-3.68) | 0.186 |  |  |  |  |
| Absent | 1.00 |  | Fibrinogen, g/L | | |  |  |  |  |
| Present | 11.49 (5.42-24.37) | ＜0.001 | ＜3.1 | 1.00 |  |  |  |  |  |
| NLR | | | ≥3.1 | 3.75 (1.89-7.43) | ＜0.001 |  |  |  |  |
| ＜2.05 | 1.00 |  | Total cholesterol, mmol/L | | |  |  |  |  |
| ≥2.05 | 2.28 (1.19-4.35) | 0.013 | ＜3.36 | 1.00 |  |  |  |  |  |
| PLR | | | 3.36-5.69 | 24622.47 (0.00-3.16E88) | 0.918 |  |  |  |  |
| ＜126.83 | 1.00 |  | ＞5.69 | 24457.93 (0.00-3.14E88) | 0.919 |  |  |  |  |
| ≥126.83 | 1.96 (1.03-3.70) | 0.040 | Triglycerides/HDL-C | | |  |  |  |  |
| MLR | | | ＜1.08 | 1.00 |  |  |  |  |  |
| ＜0.21 | 1.00 |  | ≥1.08 | 1.91 (1.02-3.57) | 0.045 |  |  |  |  |
| ≥0.21 | 1.98 (1.05-3.70) | 0.034 | Albumin, g/L | | |  |  |  |  |
| Peripheral blood eosinophils percentage, % | | | ≥42.42 | 1.00 |  |  |  |  |  |
| ＜0.7 | 1.00 |  | ＜42.42 | 2.00 (1.06-3.75) | 0.031 |  |  |  |  |
| ≥0.7 | 2.83 (0.68-11.75) | 0.152 | AST, U/L | | |  |  |  |  |
| Hemoglobin, g/L | | | ≤34 | 1.00 |  |  |  |  |  |
| ＜110 | 1.00 |  | ＞34 | 0.76 (0.18-3.14) | 0.701 |  |  |  |  |
| ≥110 | 2.05 (0.49-8.51) | 0.323 | ALT, U/L | | |  |  |  |  |
| RDW, % | | | ≤40 | 1.00 |  |  |  |  |  |
| ＜12.8 | 1.00 |  | ＞40 | 0.56 (0.14-2.34) | 0.431 |  |  |  |  |
| ≥12.8 | 1.89 (1.00-3.57) | 0.049 | ALP, U/L | | |  |  |  |  |
| Platelet count, 10^9/L | | | ≤150 | 1.00 |  |  |  |  |  |
| ＜135 | 1.00 |  | ＞150 | 0.05 (0.00-215840.31) | 0.699 |  |  |  |  |
| 135-350 | 22967.79 (0.00-2.25E144) | 0.951 | Creatinine, umol/L | | |  |  |  |  |
| ＞350 | 53490.89 (0.00-5.26E144) | 0.947 | ≤84 | 1.00 |  |  |  |  |  |
| PDW, % | | | ＞84 | 3.02 (0.72-12.59) | 0.129 |  |  |  |  |
| ＜11.5 | 1.00 |  | Uric acid, umol/L | | |  |  |  |  |
| 11.5-16.5 | 0.46 (0.11-1.98) | 0.298 | ≤420 | 1.00 |  |  |  |  |  |
| ＞16.5 | 0.64 (0.15-2.79) | 0.555 | ＞420 | 1.19 (0.37-3.88) | 0.771 |  |  |  |  |
| Blood type | | |  |  |  |  |  |  |  |
| Others | 1.00 |  |  |  |  |  |  |  |  |
| AB | 2.36 (1.04-5.39) | 0.041 |  |  |  |  |  |  |  |

**Table S6** Progression-free survival of the routine laboratory biomarkers with other clinicopathological variables
